# Chemopreventive Metabolites Are Correlated with a Change in Intestinal Microbiota Measured in A-T Mice and Decreased Carcinogenesis

**DOI:** 10.1371/journal.pone.0151190

**Published:** 2016-04-13

**Authors:** Amrita K. Cheema, Irene Maier, Tyrone Dowdy, Yiwen Wang, Rajbir Singh, Paul M. Ruegger, James Borneman, Albert J. Fornace, Robert H. Schiestl

**Affiliations:** 1 Department of Oncology, Georgetown University Medical Center, Washington, D.C., United States of America; 2 Department of Biochemistry, Molecular and Cellular Biology, Georgetown University Medical Center, Washington, D.C., United States of America; 3 Department of Environmental Health Sciences, Fielding School of Public Health, University of California Los Angeles, Los Angeles, California, United States of America; 4 Department of Biostatistics, Biomathematics and Bioinformatics, Georgetown University Medical Center, Washington, D.C., United States of America; 5 Department of Plant Pathology and Microbiology, University of California Riverside, Riverside, California, United States of America; 6 Department of Pathology Geffen School of Medicine, University of California Los Angeles, Los Angeles, California, United States of America; 7 Department of Radiation Oncology, Geffen School of Medicine, University of California Los Angeles, Los Angeles, California, United States of America; Medical University of South Carolina, UNITED STATES

## Abstract

Intestinal microbiota play a significant role in nutrient metabolism, modulation of the immune system, obesity, and possibly in carcinogenesis, although the underlying mechanisms resulting in disease or impacts on longevity caused by different intestinal microbiota are mostly unknown. Herein we use isogenic Atm-deficient and wild type mice as models to interrogate changes in the metabolic profiles of urine and feces of these mice, which are differing in their intestinal microbiota. Using high resolution mass spectrometry approach we show that the composition of intestinal microbiota modulates specific metabolic perturbations resulting in a possible alleviation of a glycolytic phenotype. Metabolites including 3-methylbutyrolactone, kyneurenic acid and 3-methyladenine known to be onco-protective are elevated in Atm-deficient and wild type mice with restricted intestinal microbiota. Thus our approach has broad applicability to study the direct influence of gut microbiome on host metabolism and resultant phenotype. These results for the first time suggest a possible correlation of metabolic alterations and carcinogenesis, modulated by intestinal microbiota in A-T mice.

## Introduction

Ataxia telangiectasia (A-T) is a recessive genetic disorder of childhood that occurs in one out of 100,000 humans worldwide. A-T is a multi-system, progressive disorder associated with a high incidence of lymphoid malignancies [1]. Approximately 30–40% of A-T patients develop neoplasia during their life and more than 40% of all tumors in A-T patients are non-Hodgkin’s lymphomas, ~20% acute lymphocytic leukemias, and 5% Hodgkin’s lymphomas [2–6]. A-T patients suffer from compromised immune function, neurodegeneration, infections of the respiratory system, and increased mortality due to 1000-fold increase in malignancy [1, 7–9]. Although ground breaking studies have led to a better and deeper understanding of ataxia telangiectasia mutated (ATM) gene function [10, 11], no effective therapy is currently available to prevent cancer or progressive neurodegeneration.

Several laboratories have generated different strains of *Atm*-deficient (*Atm*^-/-^) mice and reported that *Atm*^*-/-*^ mice developed lymphomas and died between 2 and 5 months of age [12–16]. Later studies, including our own, showed that the onset of lymphoma can be drastically delayed till 7–12 months, which was not a result of genetic diversity [17–20]. Instead, it was found to be associated with changes in intestinal microbiota, including an increase in *Lactobacillus johnsonii* [21], and reduced lymphoma incidence was achieved by administering the antioxidant N-acetyl cysteine [19]. The inflammatory effect of normal intestinal microbiota compositions could shorten lifespan of mice compared to mice housed under sterile housing conditions. Subsequently, we generated *Atm*^*-/-*^ mice harboring different but well characterized intestinal microbiota, termed conventional microbiota (CM), and restricted microbiota (RM). Restricted microbiota harbor relatively few bacterial species, dominated by unclassified *Bacteroidetes*, and are free of known pathogens [22]. RM mice were originally obtained by Caesarean section and re-derived into the mouse colony with restricted flora adoptive mothers [23]. Sterile housing and a restriction of intestinal microbiota (in RM mice) yielded a 4.5-fold lower level of genetic instability, differences in acute leukocyte genotoxicity, inflammation markers, and a 2.5-fold delayed onset of lymphoma resulting in longevity as compared to mice with CM [21]. We further demonstrated that increased levels of *L*. *johnsonii* present in RM mice, led to low basal levels of chromosomal genotoxicity and inflammation in *Atm*^*-/-*^ mice compared to the CM mice [21, 24]. Given that energy metabolism has been implicated in oncogenesis, we hypothesized that the microbiome is likely to influence host metabolism resulting in an altered phenotype. In this study, we used a comparative fecal and urine metabolomics profiling approach to discern how either of the microbiota compositions, CM or RM, would impact the metabolic phenotype of *Atm*^*-/-*^ mice.

Gut microbiome are known to have a profound influence of a myriad of diseases including dementia, obesity and cancer, although the molecular implications of dysbiosis are yet to be elucidated [25]. For example, microbiota have been shown to influence energy metabolism, beta-oxidation of lipids, bile acid, glutamine and tryptophan metabolism, as well as oxidative stress and immune response metabolites [26, 27]. Most of recently reported studies focus on the regulatory and signaling pathways that are directly affected by microbiome in different patho-physiological conditions; however, there is a lack in studies that directly examine the metabolic consequences of alterations in gut microbiome.

Metabolomics is a qualitative and quantitative evaluation of alterations in endogenous metabolism in response to a specific perturbation in a biological system [28]. It represents the endpoint of genetic regulation and its impact on altered enzymatic activities and endogenous biochemical reactions in a cell [29]; thus characterizing precise changes in metabolism is likely to enable better understanding of how cells adapt to oxidative stress and minimize DNA damage and genomic instability. Moreover, correlation of metabolic profiles with gut microbiota offers to provide valuable insights into host-microbe interactions as well as valuable steps for achieving a breakthrough in personalized therapy paradigm [30–33]. Since ATM function influences cell signaling and metabolism [34], a metabolomics approach using isogenic mouse models differing only in microbiome composition should allow for a direct assessment of accumulated bacterial metabolites and the effects of the microbiome on metabolism [35].

## Methods

### Reagents and standards

Acetonitrile (ACN), methanol, and water (all LC-MS grade) were purchased from Fisher Scientific (NJ, USA). Debrisoquine, 4-nitrobenzoic acid (4-NBA), and heparin were purchased from Sigma (USA). All standard compounds used for tandem mass spectrometry based validation of metabolite identification were bought from Sigma. Lipid standards were ordered from Avanti Polar Inc. Other reagents for preparation and analyses of samples are described in the respective sections.

### Animal housing and husbandry

The study was performed under the guidelines of the UCLA Animal Research Committee and specifically approved procedures for animal breeding, housing and sample collection in this study. Mice were housed under two types of specific pathogen free (SPF) conditions at UCLA Department of Laboratory and Animal Research, where either sterile (for RM), or non-sterile (for CM) food, water, and bedding were employed. *Atm*^*-/-*^ mice harboring RM and CM microbiota were created by rederivation as described in Fujiwara et al. [23], and by antibiotic treatment [36] followed by orogastric gavage of CM feces, respectively. The same procedures were applied in wild type mice. Mice of the RM colony were maintained under strictly aseptic and sterile conditions at the animal facility of the Radiation Oncology Department for several years. *Atm*^*-/-*^ mice were obtained by intercrossing *Atm*^*+/-*^ mice and identified by genotyping as previously described [21]. CM and RM mice for the metabolomics study were born in the respective colonies and were not treated with antibiotics. Urine and fecal samples from male *Atm*^*-/-*^ and *Atm*^*+/+*^ (referred to as wild type, or WT) mice aged 6–8 months were investigated by metabolomics-based molecular phenotyping. The different mice strains used in this study were defined as follows: *Atm*-KO-RM (Atm knock out mice carrying restricted microbiota); *Atm*-KO-CM (Atm knock out mice carrying conventional microbiota); WT-RM (wild type mice carrying restricted microbiota); and WT-CM (wild type mice carrying conventional microbiota). All urines and feces were separately collected in Eppendorf tubes from individual mice. The urine samples were centrifuged and the supernatant was aliquoted and frozen at -80°C until further use. All experiments were performed using at least 5 mice per genotype for metabolomics and at least 3–4 mice for 16S RNA sequencing experiments.

### Intestinal bacterial analysis

For this study, Illumina-based high-throughput sequencing of bacterial 16S rRNA genes from intestinal mucosa samples [37] was performed as described [38]. DNA was extracted from these samples using the PowerSoil DNA Isolation Kit (MO BIO Laboratories, Carlsbad, CA, USA), and a 30-second bead-beating step using a Mini-Beadbeater-16 (BioSpec Products, Bartlesville, OK, USA).

### Metabolite extraction and metabolomics profiling

Urine samples were processed as described previously [39] using 66% acetonitrile in water to precipitate any proteins or cellular debris. The samples were centrifuged, dried under vacuum, and stored at -80°C until analysis. Fecal samples were processed by initially sonicating in extraction solvent containing water and methanol (50% v/v) and internal standards, followed by addition of chloroform (1:1 v/v). The samples were centrifuged and the two biphasic layers were separated carefully. To each of the solvent phase two volumes of acetonitrile was added and mixed thoroughly by vortexing. The samples were incubated at -20°C for 4 hours to allow protein precipitation followed by centrifugation. The supernatant was combined and dried under vacuum, and resuspended in water containing 5% methanol for MS analysis.

Metabolites extracted from urine and fecal samples were analyzed in the same batch with two technical replicates for each sample to assess chromatographic reproducibility. The sample queue was randomized to avoid bias. Each sample (5 μl) was injected onto a reverse-phase 50 × 2.1 mm Acquity 1.7μm BEH C18 column (Waters Corp, Milford, MA), using an Acquity UPLC (Waters Corporation, USA) system online with an electrospray quadrupole time-of-flight tandem mass spectrometer (ESI-Q-TOF) (QTOF Premiere, Waters Corporation, USA). Positive and negative ion mode were operated; the details of tune page parameters have been described before [39, 40]. Together, we performed a broad range metabolite extraction and used reversed phase chromatography that would support the detection of a broad class of metabolites for urine and fecal samples. These data were normalized to the ion intensity of the internal standards (debrisoquine and 4, nitrobenzoic acid) and weight of the fecal pellet (for fecal samples), or creatinine (for urine samples). A 500 pg/μL solution of sulfadimethoxine in 50% acetonitrile ([M + H] ^+^, m/z 311.0814) was infused at 0.08 μL/min flow rate as the reference mass (lock mass) for accurate mass measurements. The quality control (QC) samples for each matrix comprised an aliquot of all samples in the study set, thus representing a universal set of metabolites. The QC sample was used to initially condition the column and thereafter, it was injected after every ten injections to account for reproducibility of the LC-MS data [41]. The coefficient of variance was examined for internal standards and creatinine in the QC samples and was found to be less than 15%. As explained above, five biological replicates per group were used for comparative profiling, while pooled QC samples were used for assessing technical reproducibility throughout each batch acquisition.

### Statistical analysis of LC-MS data and Pathway Analysis

The UPLC-QTOF raw data files were converted into NetCDF format (Network Common Data Form) using the MassLynx software (Waters Corp, Milford, MA). Subsequently, the LC-MS data were preprocessed using XCMS software as described [42]. The normalized data were processed using Metaboanalyst software (2.0) [43] and univariate analysis methods. Principal components analysis (PCA) was performed as an exploratory analysis to check for inter-group variability based on overall metabolite profiles. The candidate markers were selected by examining each volcano plot thereby considering fold change threshold of 2 and statistical p-value less than 0.05. A Benjamini-Hochberg correction was applied, which estimates conservative q-values [44], to ensure control of the false discovery rate (FDR) at a significance level of 0.05. Relative quantitation was achieved for molecular ions using the UPLC-QTOF system by taking a ratio of normalized intensity of the respective for the ions of interest in the comparative groups. This approach has been used by several laboratories including ours in previously reported studies [45–48]. The significant peaks from the FDR-adjusted analysis were putatively identified based on their m/z against Metabosearch, which performs accurate mass-based metabolite search through four main online databases; the Human Metabolome Database (HMDB), Lipid Maps, Madison Metabolomics Consortium Database (MMCD), and Metline [49]. The mass tolerance was kept at 5 parts per million to minimize false positive identification. The identity of metabolites was subsequently confirmed by comparisons of fragmentation spectra and retention time with commercially available standard compounds using tandem mass spectrometry (MS-MS). Signal intensities of the differentially abundant metabolites were visualized as a heat map, wherein the log transformed data were hierarchically clustered by Pearson correlation and average linkage clustering. The mean signal intensity is colored black, red indicates above-mean intensity, green denotes below-mean intensity and the degree of color saturation reflects the magnitude of intensity relative to the mean. Both, fecal and urine metabolomics data sets were analyzed using the Ingenuity Pathway Analysis (IPA) tool to score for correlative biochemical pathways.

## Results

### RM mice have a distinct phylotype-reduced and pathogen-depleted intestinal microbiota composition compared to CM mice

We created a mouse model with a selectively restricted microbiota, in which mice exhibit diminished T-cell immunity [37, 50], low risk for inflammatory infections, and high susceptibility to ionizing radiation in conjunction with proliferative pro-inflammatory T-cell signaling [22]. In prior research, the bacterial communities in feces from these mice were investigated by pyrosequencing of the bacterial 16S rRNA gene, and distinct profiles were identified for *Atm*^*-/-*^ and wild type mice bearing RM and CM microbiota, respectively [21, 22]. Since we have observed prolonged lifespans associated with a reduced number of B-cells in *Atm*^-/-^ RM mice [51], incidence of inflammatory non-Hodgkin B-cell lymphoma and neoplasia was suspected to be low. Consistent with these findings, pro-inflammatory *Helicobacter hepaticus*, another *Helicobacter* sp., and *Bacteroides stercoris* were less abundant or absent in RM mice compared to CM mice in mucosa samples of small intestine and colon (Table 1). Advanced Illumina-based high-throughput sequencing was newly applied to the deep sequencing of intestinal samples from WT mice. We next investigated whether an onco-protective phenotype could be associated with differentially abundant metabolites detected in RM mice as compared to CM mice.

**Table 1:** Bacterial phylotypes inhabiting the mucosa of small intestine and colon samples from CM and RM mice identified by high throughput Illumina analysis of the 16S rRNA gene.

**Nearest Cultured Relative (accession) (% identity) (Differentially abundant in intestine)**	**CM**	**RM**	**P**
Unclassified Bacteroidetes (AY239461) (94% identity)	8%	49%	0.049
*Barnesiella intestinihominis* (AB547647) (95% identity)	0%	4%	0.029
**Nearest Cultured Relative (accession) (% identity) (Differentially abundant in colon)**	**CM**	**RM**	**P**
*Helicobacter hepaticus* ATCC 51449 (NR_102911) (100% identity)	17%	0%	0.013
*Helicobacter* sp. MIT 04–8588 (GU902718) (100% identity)	11%	0%	0.002
*Bacteroides stercoris* (AB714307) (100% identity)	8%	0%	0.038
Unclassified Bacteroidetes (AY239461) (94% identity)	12%	49%	0.003
Unclassified Bacteroidetes (AY239461) (96% identity)	2%	5%	0.017

Values in CM and RM columns are % of Illumina rRNA gene reads from intestinal mucosal samples. These values were compared by 2-tailed Student’s T-Tests. n = 3–4 mice for each of the two colonies. CM is conventional microbiota. RM is restricted microbiota. % identity was determined by analyses using Blast (NCBI) [69].

### Microbiota drive changes in urinary metabolomics profiles in Atm^-/-^ and WT mice

**Fig 1:**
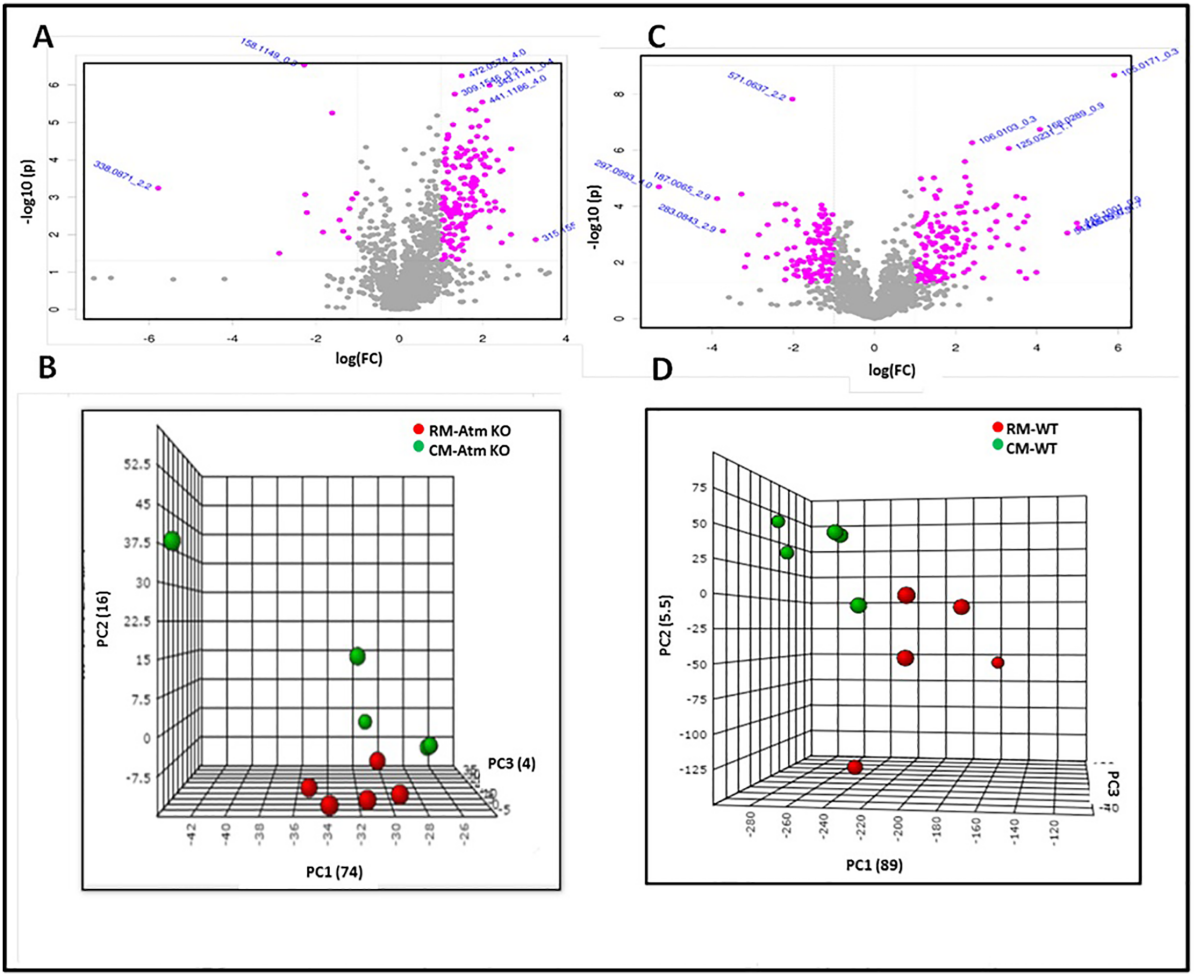
Urine metabolomics in RM and CM mouse models. Panel A and C. Volcano plots facilitating visualization of differentially abundant metabolites that were selected based on fold change (X-axis) and p-value in (Y-axis) for Atm-KO and WT mice, respectively. The m/z values highlighted in pink have a fold change of ≥ 0.5 or ≤ 2.0 and p-value ≤ 0.05 in RM as compared to CM mice and were selected for further characterization. Panel B and D: PCA plots showing separation between RM and CM in Atm-KO and WT mice, respectively.

**Fig 2:**
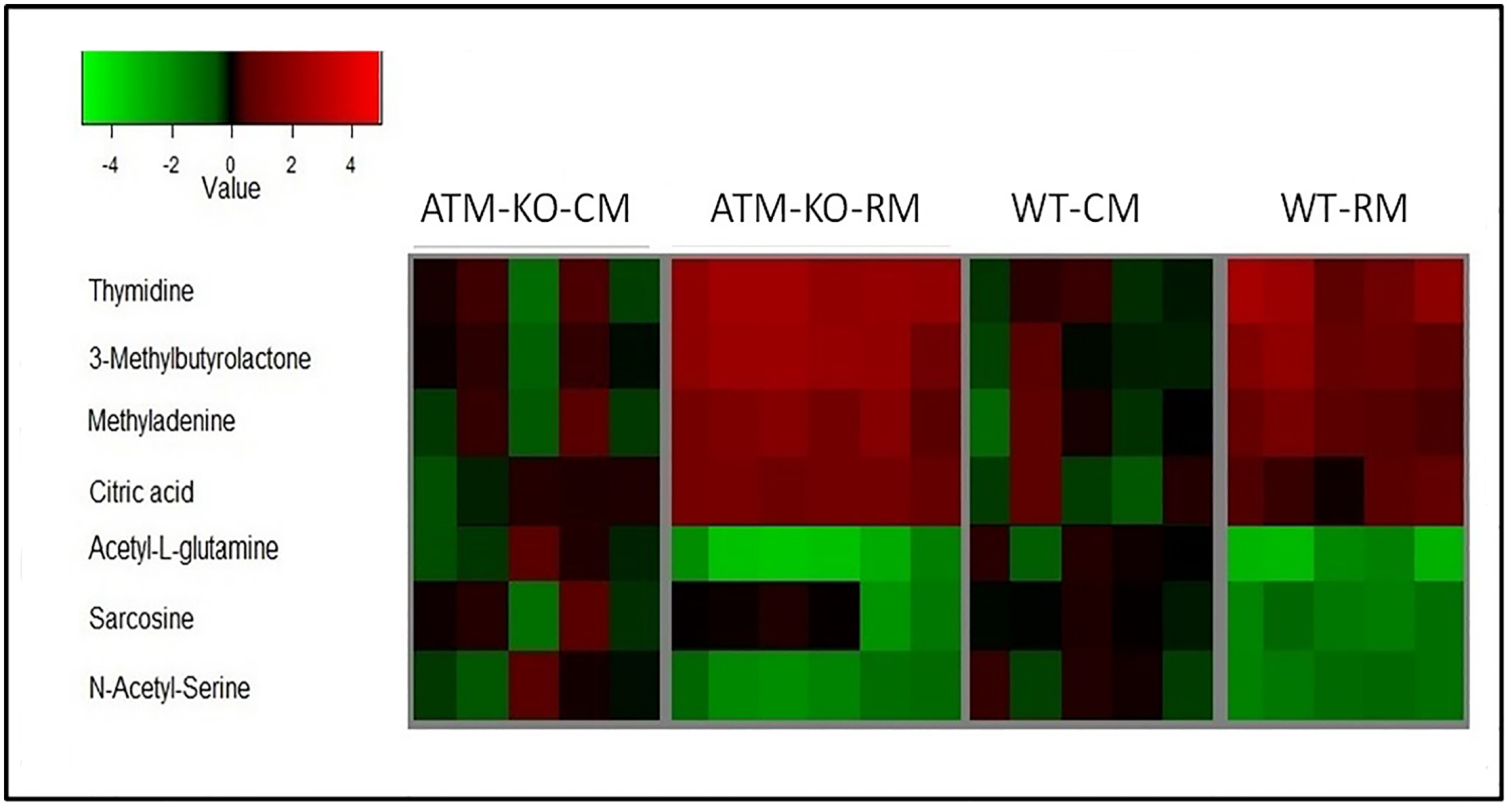
Heat map showing differential abundance of urine metabolites in the various study groups: Metabolites profiles for Atm-KO-CM group, Atm-KO-RM, WT-CM, and WT-RM, respectively. Each column represents a sample, and each row represents a metabolite. The mean signal intensity of CM group is colored black; red indicates above-mean intensity, green denotes below mean intensity, and the degree of color saturation reflects the magnitude of intensity relative to the mean.

To investigate microbiota-associated metabolic alterations, we performed comparative metabolomic profiling of urine samples obtained from *Atm*^*-/-*^ or *Atm*^*+/+*^ (wild type, or WT) mice harboring either the RM or CM microbiota. Pre-processing using XCMS resulted in a three dimensional data matrix (m/z, retention time, and intensity values) consisting of a total of 1588 (*Atm*-KO-RM), 1672 (*Atm*-KO-CM), 1465 (WT-RM) and 1575 (WT-CM) features that were subsequently used for statistical analyses. Candidate markers were selected by examining the volcano plot and considering a fold change threshold of 2 and p-value less than 0.05 (Fig 1, Panels A and C). Principal component analysis resulted in a reasonable class separation of *Atm*-KO and WT groups harboring CM or RM microbiota, respectively (Fig 1, Panels B and D). The identity of some metabolites was confirmed using tandem mass spectrometry (Table 2) and these are visualized as a heat map (Fig 2). For some other metabolites that came up as being significantly dysregulated on the volcano plots we were unable to find biological annotations in the databases. Interestingly, the presence of RM microbiota influenced both genotypes in a similar manner, although changes in the levels of urinary citrate were not significant for the WT mice. The urinary levels of 3-methylbutyrolactone were found to be high in *Atm*-KO-RM and WT-RM mice. Additionally, mice with RM microbiota also showed elevation of methyladenine, while the levels of metabolites like sarcosine, N-acetyl serine, and N-acetyl glutamine were depleted in mice of both genotypes with RM as compared to CM mice. Additionally, *Atm*-KO-RM mice showed upregulation of metabolites participating in TCA cycle like aconitic acid, and substituted amino acids as intermediary metabolites of purine like nicotinate D-ribonucleoside (Table 3). WT-RM mice showed an increase in the urinary levels of N-phosphoacetyl-L-asparatic acid; a metabolite that has been shown to have a strong anti-tumor activity (Table 4) [52]. Both genotypes of RM mice showed differential abundance of a number of di- and tripeptides differing in composition.

**Table 2:** Validated urine metabolites that were differentially abundant in RM as compared to CM in Atm-KO and WT mice.

Name of the Metabolite	ESI Mode	m/z*	RT **	Atm KO			WT			Major CID Fragments
				FC***	p-value	q-value	FC***	p-value	q-value	
Thymidine	POS	243.0998	4.0	**↑**	<0.0001	0.0001	**↑**	0.0062	0.0129	170.0746, 172.0893
3-Methylbutyrolacetone	POS	101.0603	1.2	**↑**	0.0001	0.0009	**↑**	0.0070	0.0131	55.05550, 73.0661, 83.0524
Methyladenine	POS	150.0778	0.4	**↑**	0.0011	0.0037	**↑**	0.0166	0.0249	108.0446, 123.0674
Citric acid	NEG	191.0182	0.3	**↑**	<0.0001	0.0001	**↑**	0.0937	0.1125	85.0291, 87.0086, 111.0085
Acetyl-L-glutamine	NEG	187.0717	0.3	**↓**	0.0001	0.0009	**↓**	<0.0001	0.0002	125.0736, 145.0646
Sarcosine	POS	90.0541	1.1	**↓**	0.4276	0.4528	**↓**	<0.0001	0.0001	72.04
N-Acetyl-Serine	NEG	146.0447	0.3	**↓**	0.0012	0.0039	**↓**	0.0006	0.0026	74.252, 84.0457, 98.0257

* m/z = Mass/charge;

** RT = Retention Time in minutes;

*** FC = Fold Change = RM/CM (≤ 0.5 or ≥ 2.0);

q-value = False Discovery Rate (FDR) adjusted p-value.

**Table 3:** Urine metabolites that were differentially abundant in RM as compared to CM in only Atm-KO mice.

Metabolite Name	ESI Mode	m/z*	RT **	Atm KO (Urine)	
				FC***	p-value
Aconitic acid	NEG	173.0078	0.4	**↑**	0.0001
N-Acetylornithine	POS	175.1092	2.6	**↑**	0.0024
O-Ureidohomoserine	POS	178.0811	2.5	**↑**	0.0050
Asn Asp	POS	248.0872	2.8	**↑**	0.0006
Nicotinate D-ribonucleoside	POS	256.0797	0.4	**↑**	0.0141
Oleamide	POS	282.2785	8.0	**↓**	0.0316
Glycylprolylhydroxyproline	POS	286.1373	0.4	**↑**	0.0345
Asp Gly Cys	POS	294.0773	0.9	**↑**	0.0061
5-Thymidylic acid	POS	307.0716	0.5	**↑**	0.0154
Octanoylglucuronide	NEG	319.1394	2.5	**↑**	0.0276
Thr Thr Thr	POS	322.1586	4.8	**↑**	0.0151
Phe Ala Asn	NEG	349.1504	3.7	**↑**	0.0495
Ala His Glu	NEG	354.1398	4.9	**↓**	0.0004
1-Phosphatidyl-D-myo-inositol	NEG	389.0529	1.9	**↓**	0.0023
Met Ser Arg	POS	393.1943	2.7	**↑**	0.0010
Lys-Phe-OH	NEG	400.1501	1.0	**↑**	0.0042
Trp-Asp-OH	POS	442.1236	4.1	**↑**	0.0006
Aspartyl-2-deoxy-adenosine-5-monophosphate	POS	447.0988	3.5	**↑**	0.0019
serine hydroxamate-AMP	NEG	448.0999	3.6	**↑**	0.0057
Trp-Phe-OH	NEG	472.1521	3.7	**↓**	0.0098
Arachidonoyl PAF C-16	POS	768.5948	9.3	**↑**	0.0453
GPCho(18:2/18:2)	NEG	781.5600	5.1	**↓**	0.0038

* m/z = Mass/charge;

** RT = Retention Time in minutes;

*** FC = Fold Change = RM/CM (≤ 0.5 or ≥ 2.0).

**Table 4:** Urine metabolites that were differentially abundant in RM as compared to CM in only WT mice.

Metabolite Name	ESI Mode	m/z*	RT **	WT (Urine)	
				FC***	p-value
Dihydroxyquinoline	POS	162.0544	2.0	**↓**	0.0005
Hydroxyadipic acid	POS	163.0604	2.2	**↓**	0.0004
m-Hydroxyhippuric acid	NEG	194.0452	2.8	**↓**	0.0005
Dopaquinone	NEG	194.0470	0.6	**↑**	0.0002
Decenedioic acid	NEG	199.0984	4.6	**↓**	0.0261
Pantothenic Acid	POS	220.1173	1.1	**↑**	0.0000
N-acetyl-beta-D-glucosaminylamine	POS	221.1116	1.1	**↓**	0.0000
N-octanoyl-L-Homoserine lactone	NEG	226.1432	5.4	**↓**	0.0220
Ser Lys	POS	234.1455	4.2	**↓**	0.0254
Tiglylcarnitine	NEG	242.1391	4.9	**↓**	0.0176
N-phosphonoacetyl-L-ornithine	NEG	253.0608	2.9	**↓**	0.0146
N-phosphonoacetyl-L-aspartic acid	NEG	254.0061	1.6	**↑**	0.0061
Asn Gln	NEG	259.1026	4.5	**↓**	0.0004
3-Methoxy-4-hydroxyphenylglycol sulfate	NEG	263.0237	0.8	**↑**	0.0298
Ala Ala Lys	NEG	287.1707	4.9	**↓**	0.0473
Thr Ala Thr	POS	292.1512	4.2	**↓**	0.0229
2-Oxo-nonadecanoic acid	POS	313.2717	8.1	**↓**	0.0001
Gly Met Asn	POS	321.1234	3.9	**↓**	0.0409
Cys Asp Cys	NEG	338.0512	1.1	**↓**	0.0033
Val Thr Glu	NEG	346.1650	5.8	**↓**	0.0198
Thr-Asp-OH	NEG	355.0807	4.0	**↑**	0.0000
Asn Ser His	NEG	355.1383	5.0	**↓**	0.0067
Cys Val His	NEG	356.1388	5.0	**↓**	0.0050
Cys Pro Phe	POS	366.1511	2.1	**↓**	0.0005
Ala Tyr Asn	POS	367.1609	2.1	**↓**	0.0004

* m/z = Mass/charge;

** RT = Retention Time in minutes;

*** FC = Fold Change = RM/CM (≤ 0.5 or ≥ 2.0).

### Microbiota drive alterations in the fecal metabolome in Atm^-/-^ and WT mice

**Fig 3:**
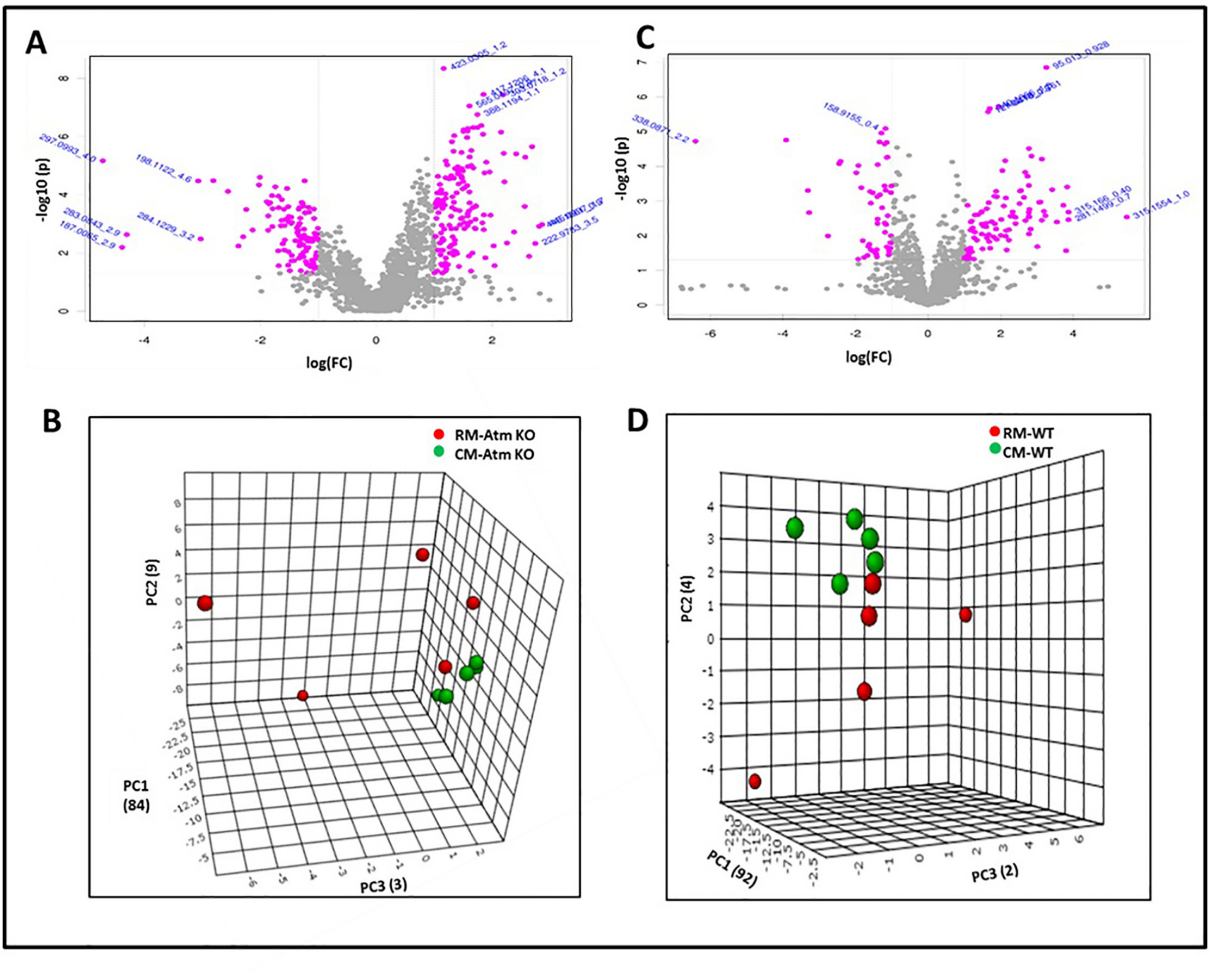
Gut microbiota modulates fecal metabolomic profiles in Atm-KO and WT mice. Panel A and C. Volcano plots facilitating visualization of differentially abundant metabolites that were selected based on fold change (X-axis) and p-value in (Y-axis) for Atm-KO and WT mice, respectively. The m/z values highlighted in pink have a fold change of ≥ 0.5 or ≤ 2.0 and p-value ≤ 0.05 in RM as compared to CM mice and were selected for further characterization. Panels B and D: PCA plots showing separation between RM and CM in Atm-KO and WT mice, respectively.

**Fig 4:**
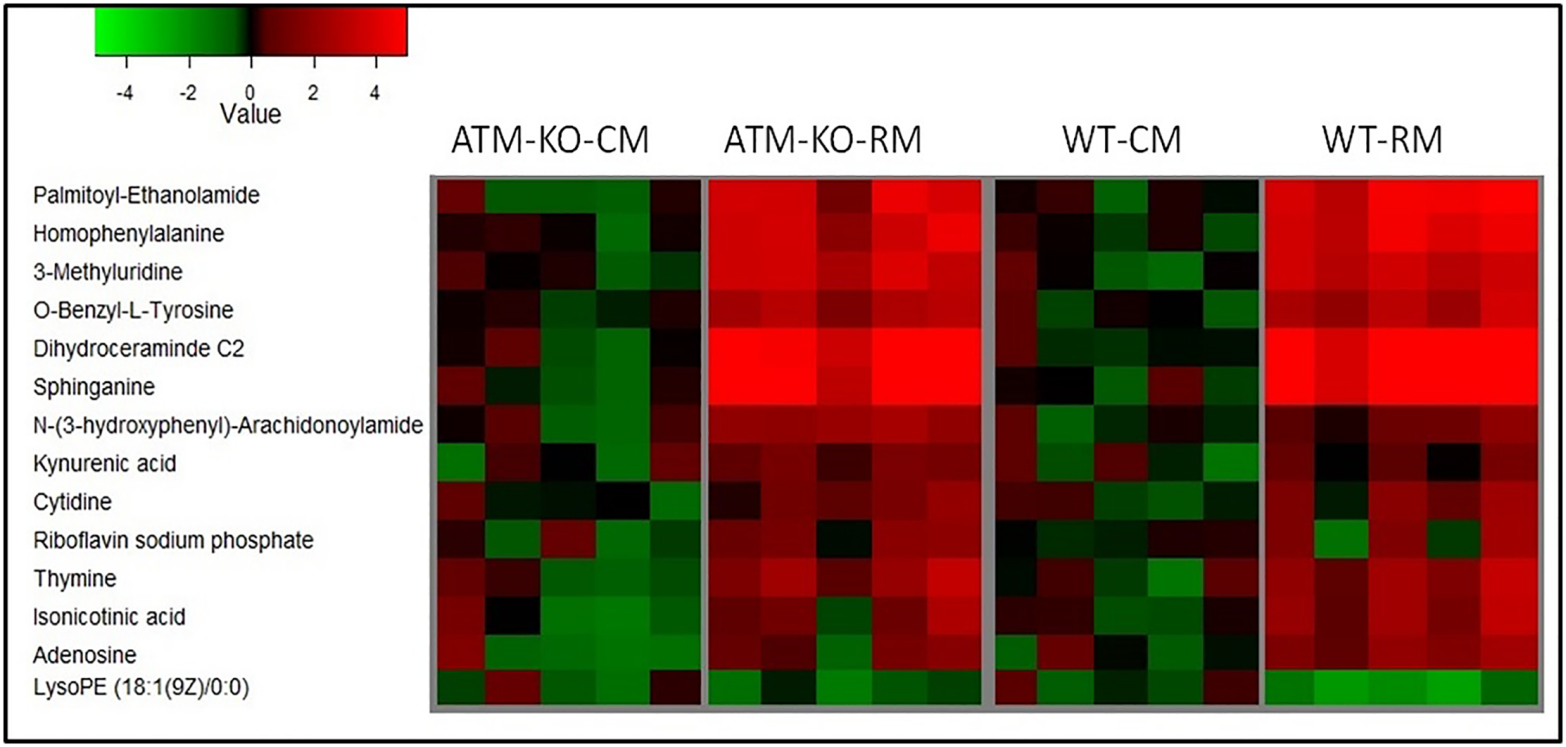
Heat map showing differential abundance of fecal metabolites in various study groups (Panels A-D): Metabolites profiles for Atm-KO-CM group, Atm-KO-RM, WT-CM, and WT-RM, respectively. Each column represents a sample, and each row represents a metabolite. The mean signal intensity of CM group is colored black; red indicates above-mean intensity, green denotes below mean intensity, and the degree of color saturation reflects the magnitude of intensity relative to the mean.

Next we performed metabolomic profiling of fecal samples obtained from *Atm*^*-/-*^ or wild type (WT) mice harboring either the RM or CM microbiota. Pre-processing using XCMS resulted in 1478 (*Atm*-KO-RM), 1742 (*Atm*-KO-CM), 1655 (WT-RM) and 1675 (WT-CM) features that were subjected to multivariate analyses. Putative markers were selected by examining the volcano plot and considering a fold change threshold of 2 and p-value less than 0.05 (Fig 3, Panels A and C). Again, principal component analysis resulted in clear separation of *Atm*^*-/-*^ and WT groups harboring CM or RM microbiota, respectively (Fig 3, Panels B and D). The identity of these metabolites was confirmed using tandem mass spectrometry (Table 5). Metabolites identified from individual difference detection analyses were visualized as a heat map (Fig 4). *Atm*-KO and WT-RM mice showed statistically significant upregulation of a number of metabolites including homophenylalanine, sphinganine, methyluridine and riboflavin sodium phosphate. There was an elevation in the levels of kynurenic acid (a tryptophan metabolite) [53]. RM mice also showed elevation in the fecal levels of riboflavin phosphate (Vitamin B2), which is a precursor of flavin adenine dinucleotide (FAD) and flavin adenine mononucleotide (FMN) that are essential cofactors of TCA cycle. In addition, *Atm*-KO-RM mice showed alterations in relative abundance of free fatty acids, secondary bile acids as well as tripeptides as compared to the *Atm*-KO-CM mice (Table 6). WT-RM mice showed differentials levels of a number of glycerophospholipids, di- and tripeptides as well as fatty acids (Table 7).

**Table 5:** Validated fecal metabolites that were differentially abundant in RM as compared to CM in the Atm-KO and WT mice.

Metabolite Name	ESI Mode	m/z*	RT **	Atm KO			WT			Major CID Fragments
				FC***	p-value	q-value	FC***	p-value	q-value	
Palmitoyl-Ethanolamide	POS	300.2896	2.5	**↑**	0.0053	0.0119	**↑**	0.0083	0.0131	62.0612, 95.0872
Homophenylalanine	POS	180.1006	0.9	**↑**	0.0046	0.0113	**↑**	0.0007	0.0031	97.0512, 117.0698
3-Methyluridine	NEG	257.0775	0.4	**↑**	0.0003	0.0013	**↑**	<0.0001	0.0003	82.03001, 154.0508,
										214.0718
O-Benzyl-L-Tyrosine	POS	272.1296	0.4	**↑**	0.0009	0.0033	**↑**	0.006	0.0129	91.0553, 226.1236, 255.1023
Dihydroceramide C2	POS	344.3183	3.1	**↑**	0.0023	0.007	**↑**	0.0078	0.0131	81.0711, 95.0867, 266.2828
Sphinganine	POS	302.3049	2.5	**↑**	0.0011	0.0037	**↑**	0.008	0.0131	91.0552, 254.2855
3-hydroxyphenyl-Arachidonoyl amide	POS	396.2922	3.7	**↑**	<0.0001	0.0003	**↑**	0.0601	0.0738	110.0616, 203.1800
Kynurenic acid	NEG	188.0365	1.7	**↑**	0.0171	0.025	**↑**	0.1489	0.1711	144.044
Cytidine	NEG	242.0764	1.2	**↑**	0.0429	0.0538	**↑**	0.0405	0.0521	109.0395
Riboflavin sodium phosphate	NEG	477.0794	0.3	**↑**	0.0261	0.0371	**↑**	0.1368	0.1606	78.9596, 96.9696
Thymine	POS	127.0495	0.6	**↑**	0.031	0.043	**↑**	0.0391	0.0515	84.0450, 110.0238
Isonicotinic acid	POS	124.0405	0.4	**↑**	0.1739	0.1956	**↑**	0.0322	0.0434	80.0504, 96.0450,
										124.0398
Adenosine	POS	268.1035	0.6	**↑**	0.2196	0.2371	**↑**	0.0082	0.0131	94.0413, 136.0631
LysoPE(18:1(9Z)/0:0)	NEG	478.2967	3.5	**↓**	0.2118	0.2334	**↓**	0.0045	0.0113	78.9599, 281.2458

* m/z = Mass/charge;

** RT = Retention Time in minutes;

*** FC = Fold Change = RM/CM (≤ 0.5 or ≥ 2.0);

q-value = False Discovery Rate (FDR) adjusted p-value.

**Table 6:** Fecal metabolites that were differentially abundant in RM as compared to CM only in Atm-KO mice.

Metabolite Name	ESI Mode	m/z*	RT **	Atm KO (Fecal)	
				FC***	p-value
L-Methionine	NEG	148.0432	0.4	**↓**	0.0014
Tryptophan	POS	205.0965	1.6	**↓**	0.0374
Hydroxydecanedioic acid	NEG	217.1082	2.4	**↓**	0.0231
6-Acetyl-D-glucose	NEG	221.0687	1.5	**↓**	0.0053
9,12-Dioxo-dodecanoic acid	NEG	227.1280	3.4	**↓**	0.0357
Malonylcarnitine	POS	248.1122	0.4	**↓**	0.0078
AFMK (Acetyl-N-formyl-5-methoxykynurenamine)	NEG	263.1011	2.3	**↓**	0.0035
Heptanoylcarnitine	NEG	272.1844	2.9	**↓**	0.0068
trans-Farnesyl phosphate	NEG	301.1586	3.1	**↓**	0.0243
Ser Arg Gly	NEG	317.1577	2.9	**↓**	0.0213
Ser Lys Ser	NEG	319.1647	3.0	**↓**	0.0044
Met Val Ala	POS	320.1669	2.9	**↓**	0.0299
Phe Arg	NEG	320.1700	3.0	**↓**	0.0050
Eicosapentaenoyl ethanolamide	POS	346.2734	3.1	**↓**	0.0237
Cys Gln Cys	POS	353.0977	2.7	**↓**	0.0143
DHA ethyl ester	POS	357.2794	4.3	**↓**	0.0465
3-Oxo-chol-11-enic acid	POS	373.2752	3.8	**↓**	0.0425
Ile Thr Phe	POS	380.2167	7.5	**↓**	0.0465
Hydroxytetracosenoic acid	NEG	381.3370	8.4	**↑**	0.0158
His Asp Asn	NEG	383.1299	2.8	**↓**	0.0002
Allochenodeoxycholic acid	NEG	391.2852	4.3	**↓**	0.0175
Vitamin D6	POS	411.3633	6.7	**↑**	0.0474
Lys His Met	POS	415.2148	4.8	**↓**	0.0257
Lys-Tyr-OH	NEG	416.1451	1.5	**↓**	0.0239
PA(17:1/0:0)	POS	423.2508	7.9	**↓**	0.0269
His Phe Met	NEG	432.1713	1.1	**↓**	0.0127
PE(P-16:0/0:0)	NEG	436.2817	6.0	**↑**	0.0283
Met Met Arg	POS	437.1989	4.8	**↓**	0.0268
PC(P-14:0/0:0)	NEG	450.3013	3.3	**↓**	0.0171
(22R)-1a,22,25-Trihydroxy-23;24-tetradehydro-24a,24b-dihomo-20-epivitamin D3	NEG	455.3139	5.3	**↓**	0.0239
Trp Trp Ala	POS	462.2094	3.8	**↓**	0.0222
PA(22:2(13Z,16Z)/0:0)	NEG	489.2991	3.2	**↓**	0.0050
Trp Asp Trp	POS	506.2044	4.0	**↓**	0.0239
D-Urobilinogen	POS	591.3198	3.1	**↓**	0.0100
Atalanine	POS	611.2065	9.3	**↓**	0.0006
PA(P-16:0/15:1(9Z))	POS	617.4588	8.2	**↓**	0.0067
Cer(d18:0/24:0)	POS	652.6553	9.0	**↓**	0.0186
PG(14:1(9Z)/14:1(9Z))	POS	663.4281	8.2	**↓**	0.0050
Palmitoyl thio-PC	POS	750.5451	9.4	**↓**	0.0484
PA(18:1/22:4)	POS	751.5342	9.4	**↓**	0.0475
MGDG(18:2/18:3)	POS	777.5552	9.4	**↓**	0.0426
PI(P-16:0/16:1)	POS	793.5189	8.3	**↓**	0.0035
16:2-Glc-Campesterol	POS	797.6345	8.9	**↓**	0.0103
PG(18:1/20:2)	POS	801.5637	8.9	**↓**	0.0049
PC(16:1/22:6)	POS	804.5513	8.0	**↓**	0.0488

* m/z = Mass/charge;

** RT = Retention Time in minutes;

*** FC = Fold Change = RM/CM (≤ 0.5 or ≥ 2.0).

**Table 7:** Fecal metabolites that were differentially abundant in RM as compared to CM only in WT mice.

Name of Unique Metabolite ID	ESI Mode	m/z*	RT **	WT (Fecal)	
				FC***	p-value
Alanine	POS	90.0550	0.3	**↓**	0.0037
3-Amino-alanine	POS	106.0743	8.5	**↑**	0.0428
Hydroxybenzoquinone	POS	125.0230	0.4	**↓**	0.0283
4-Hydroxyquinoline	NEG	144.0448	2.5	**↓**	0.0036
Orotic acid	NEG	155.0106	0.4	**↓**	0.0110
Acetyl-DL-Valine	NEG	158.0810	1.5	**↓**	0.0232
2-Hydroxylamino-4,6-dinitrotoluene	NEG	212.0314	0.4	**↓**	0.0020
3-Methoxytyrosine	POS	212.0930	1.3	**↓**	0.0043
N-Acetyl-beta-D-glucosaminylamine	POS	221.1112	1.4	**↓**	0.0438
L-4-Hydroxy-3-methoxy-a-methylphenylalanine	POS	226.1071	1.5	**↓**	0.0118
Gamma-glutamyl-proline	NEG	242.1136	2.3	**↓**	0.0238
Palmitic acid	NEG	255.2318	7.4	**↓**	0.0176
Asp Gln	POS	262.1058	0.4	**↓**	0.0217
AFMK (Formyl-N-acetyl-5-methoxykynurenamine)	POS	265.1202	2.3	**↓**	0.0316
N-Acetyl-L-glutamate 5-phosphate	NEG	268.0202	0.3	**↓**	0.0018
Isovalerylglucuronide	NEG	277.0919	1.3	**↑**	0.0068
2-methyl-16-heptadecenoic acid	NEG	281.2480	7.5	**↓**	0.0374
Met Gly Ser	NEG	292.0972	0.5	**↓**	0.0019
C20:4n-2;6;9;12	NEG	303.2316	7.0	**↓**	0.0484
Arachidic acid	NEG	311.2976	8.4	**↓**	0.0106
3-Oxo-nonadecanoic acid	POS	313.2747	7.0	**↓**	0.0010
His Gly Cys	POS	316.1097	0.6	**↓**	0.0258
Ser Pro Asp	NEG	316.1165	0.6	**↑**	0.0161
Leu Trp	POS	318.1815	2.4	**↓**	0.0433
Hydroxysphinganine	POS	318.3007	4.9	**↑**	0.0122
His Leu Gly	POS	326.1834	1.7	**↓**	0.0130
Docosanoic acid	NEG	339.3246	8.8	**↓**	0.0428
2,3-dinor Thromboxane B1	NEG	343.2122	2.9	**↑**	0.0345
Leu Thr Leu	NEG	344.2169	2.9	**↑**	0.0099
18-Oxo-resolvin E1	NEG	347.1878	3.2	**↓**	0.0391
Coutaric acid	NEG	348.1907	3.2	**↓**	0.0400
S-(2-Hydroxyethyl)glutathione	NEG	350.1028	0.5	**↑**	0.0177
MG(0:0/18:3/0:0)	NEG	351.2510	8.0	**↓**	0.0012
13,14-Dihydro PGF-1a	NEG	357.2618	3.7	**↑**	0.0211
His Asn Cys	NEG	371.1180	2.7	**↓**	0.0307
Pro Glu Met	NEG	374.1379	7.4	**↓**	0.0302
Pro His Glu	NEG	380.1610	7.0	**↓**	0.0434
Lys-Lys-OH	NEG	381.1748	7.5	**↓**	0.0461
Pentacosanoic acid	NEG	381.3723	9.5	**↓**	0.0369
Val Glu His	NEG	382.1763	7.5	**↓**	0.0456
S-Adenosylhomocysteine	NEG	383.1160	0.5	**↑**	0.0014
Glu Phe Val	POS	394.1940	2.0	**↓**	0.0232
Hexacosanoic acid	NEG	395.3859	9.9	**↓**	0.0415
PC(O-12:0/0:0)	NEG	424.2795	3.3	**↓**	0.0003
Glu Arg Glu	NEG	431.1883	4.8	**↓**	0.0077
Palmityl myristoleate	NEG	449.4324	10.6	**↓**	0.0452
PE(O-18:0/0:0)	POS	468.3489	6.6	**↑**	0.0054
Sulfocholic acid	NEG	487.2420	3.9	**↓**	0.0111
Hydroxydydrogesterone glucuronide	NEG	489.2540	3.9	**↓**	0.0109
Glycochenodeoxycholate 7-sulfate	NEG	528.2599	2.3	**↓**	0.0269
Trp Arg Trp	NEG	545.2651	4.1	**↓**	0.0228
DG(16:0/16:0/0:0)	POS	569.5133	9.4	**↑**	0.0031
Ceramide (d18:0/20:0)	POS	596.5966	10.6	**↑**	0.0451
PG(O-16:0/12:0)	NEG	651.4610	7.9	**↓**	0.0037
PG(14:0/14:0)	NEG	665.4351	7.5	**↓**	0.0318
Ceramide (t18:0/24:0)	POS	668.6561	10.1	**↑**	0.0297

* m/z = Mass/charge;

** RT = Retention Time in minutes;

*** FC = Fold Change = RM/CM (≤ 0.5 or ≥ 2.0).

**Fig 5:**
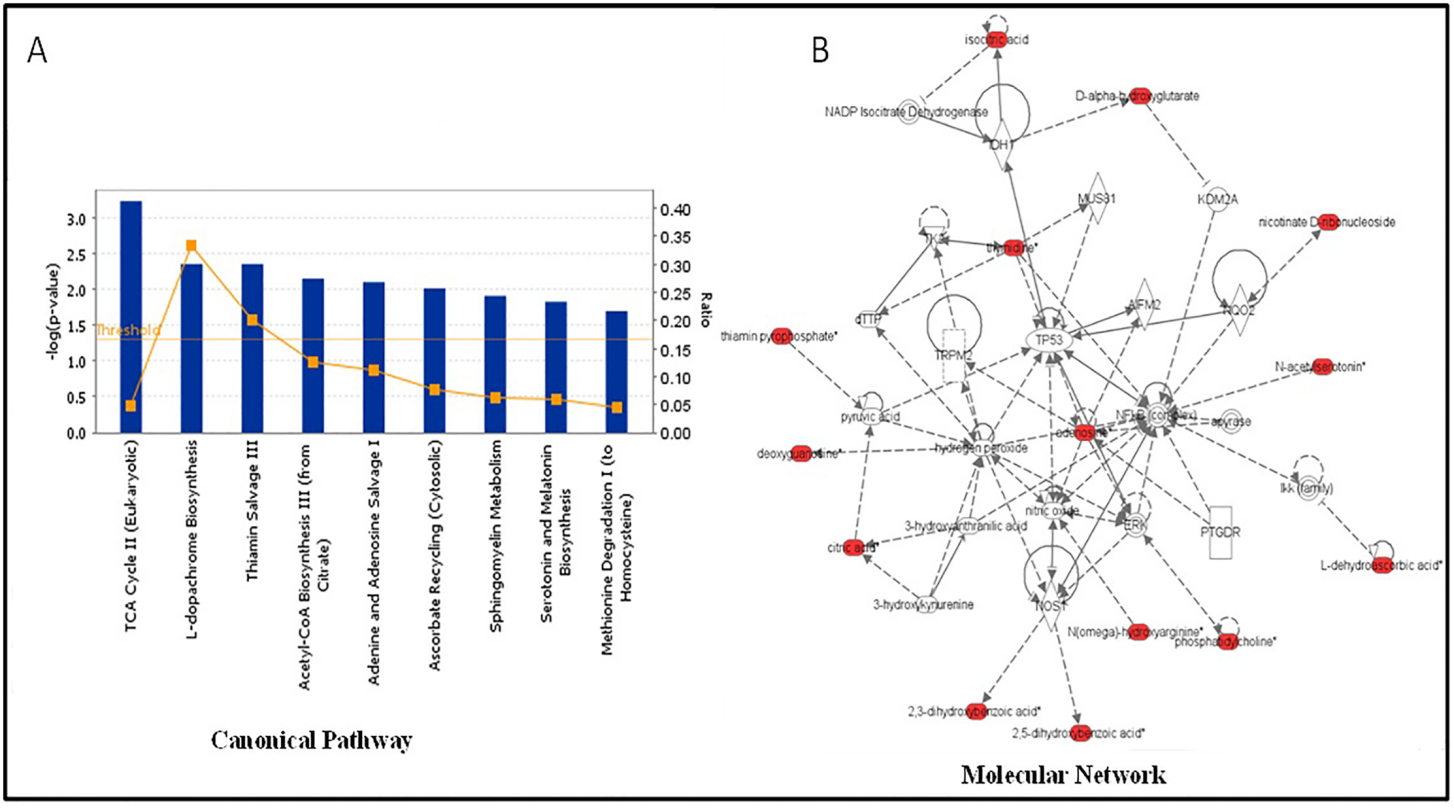
Functional Pathway Analysis showing major upregulated pathways in Atm-KO mice with RM as compared to CM microbiota. Panel A shows significantly perturbed canonical pathways in Atm-KO-RM mice, while Panel B shows a TP53 regulated network that correlated strongly with metabolic profiles of Atm-KO-RM mice.

In order to gain insights into metabolite enrichment representing specific molecular networks and pathway perturbations caused by changes in gut microbiome in *Atm*- KO mice, we used the Ingenuity Pathway Analysis (IPA) tool. This was achieved by integrating the fecal and urine metabolomics data sets that were initially scored separately for differentially expressed metabolites. Pathway analysis showed positive correlation for metabolic intermediates of TCA cycle in RM as compared to CM in *Atm*-KO mice (Fig 5 Panel A). Examination of molecular networks revealed an upregulation of TP53 regulated metabolite networks (Fig 5 Panel B). Although these networks may affect a broad range of cellular processes, they can be considered distinct key metabolites that help explain the observed phenotype in these mice. Further investigations into more specific metabolite changes may answer to the basic question of precisely how do changes in microbiota composition result in an onco-protective phenotype.

## Discussion

Despite increasing efforts in the field of A-T research, the mechanisms by which enteric microbiota lead to changes in *Atm*-mediated lymphomagenesis remain elusive. ATM regulates the cellular response to oxidative stress and it senses double-stranded DNA breaks, thus inhibiting cell cycle progression [11, 54]. It has been demonstrated that intestinal microbiota can induce cancer development [35], for example *Helicobacter pylori*, inducing gastric cancer and lymphoma in WT mice and humans, as well as potentially influencing the response of cancer to therapies [55, 56]. We have previously shown reduction of cancer susceptibility and an impact on longevity by specific alterations in gut microbiota composition in *Atm*^*-/-*^ mice. Using an untargeted, high resolution mass spectrometry approach, we then asked how this translates into metabolic changes in mice that are genetically identical but differ only in their gut microbiome composition. Surprisingly, we did not find differently abundant metabolites in blood profiles (data not shown). Moreover, our results from the analysis of urine and fecal samples suggest that distinct intestinal microbiota cause a metabolic shift towards the upregulation of metabolites including kyneurenic acid, methyladenine and 3-methybutyrolactone that may attenuate cancer-promoting signaling pathways, reported to be associated with an onco-protective phenotype independent of the genotype of the mice. We found that intestinal microbiota restriction in Atm-deficient mice led to a 2.5-fold extension of lymphoma latency and 4 fold increased longevity, and significant differences in chromosomal genotoxicity, oxidative DNA damage and inflammation; our research was the first to show a relationship between intestinal microbiota and lymphoma onset [21]. *Lactobacillus johnsonii*, one of the indicator phylotypes that we identified to be more abundant in RM than CM mice, is known to increase the number of Paneth cells, which are a host cell type that produces antimicrobial compounds, and are located together with stem cells in the intestinal crypt [57]. The extended lifespans of our RM mouse models appear to be caused by an intestinal microbiota comprised largely of unclassified members of the Bacteroidetes (Table 1), which in turn result in specific metabotypes.

Potentially chemopreventive metabolites were found to be differentially abundant in a microbiota-dependent manner in *Atm*-KO or WT mice; for example, the urinary levels of 3-methylbutyrolactone were elevated in both genotypes harboring RM microbiota. This metabolite is known to be produced by the intestinal bacteria and exhibits strong anti-aromatase properties [58]. Further, this metabolite inhibits fatty acid synthase (FAS), which activates carnitine palmitoyltransferase 1beta (CPT-1beta) and ultimately results in beta-oxidation of long chain fatty acids and prevention of lipid accumulation [59]. In addition, alpha-methylbutyrolactone acts as a selective alkylating agent for thiol rich enzymes like phosphofructokinase, DNA polymerase and glycogen synthase; thus regulating cell proliferation [60]. Additionally, mice with RM microbiota showed elevation of methyladenine, which is a potent inhibitor of phosphoinositide 3-kinases (PI3K) and has also been shown to exhibit tumor suppressor activity [61–64]. There was an elevation in the levels of kynurenic acid (a tryptophan metabolite) in feces of RM mice. Walczak et al. have reported the potential chemopreventative role of this metabolite in colon cancer [53]. Elevation of kynurenic acid has been shown to positively correlate with immune tolerance [65–67]. An upregulation of metabolites participating in oxidative phosphorylation was found in RM as compared to CM in *Atm*- KO (Fig 5, Panel A) mice, as well as an upregulation of TP53 regulated molecular networks in RM mice (Fig 5, Panel B). Interestingly, TP53 is known to regulate the expression of TIGAR (TP53-induced Glycolysis and Apoptosis Regulator) that has been shown to downregulate glycolysis by lowering the endogenous levels of fructose 1,6 biphosphate [68]. This is an interesting finding, generating new hypothesis for investigating how alleviation of glycolytic phenotype in RM mice is induced. Future investigations in our laboratory will specifically follow the molecular links and associations proposed herein.

Our results demonstrate that microbiome composition leads to specific changes in overall host metabolism, which may have direct implications on phenotype. This proof of principle investigation opens up several relevant and challenging questions; particularly, how lipids measured in feces of these mice indirectly regulate apoptosis, or if the altered metabolic profile accounts at least in part for extended life and decreased incidence of carcinogenesis in eukaryotes and will be a part of future studies. Further investigation is also needed to specify the regulatory role of certain microbiota compositions in permitting genotoxicity by reducing local or systemic autophagy.

In conclusion, our pilot study demonstrates metabolite profiles likely modulated by specific phylotypes in RM mice that correlate with our previously reported observations of decreased tumor incidence and general leukocyte genotoxicity in RM mice as compared to *Atm*-KO-CM mice [21]. These alterations in the mean abundance of the interrogated metabolites, including 3-methylbutyrolactone and 3-methyadenine (Fig 2) and kynurenic acid (Fig 4) were more pronounced in *Atm*-KO mice as compared to the WT RM mice, which may help explain genotype based susceptibility. Together, these findings lend credence to the notion that manipulating microbial composition could be used as an effective strategy to prevent or alleviate cancer susceptibility. The onco-protective effect of RM microbiota is true for WT as well as *Atm*-KO mice—the only difference lies in cancer susceptibility for both genotypes. Remarkably, our findings suggest that composition of the gut microbiota influences and alters central carbon metabolism in a genotype independent manner. In future, it is our hope that the use of probiotics-containing RM would be a potential chemopreventive for normal humans, while the same type of microbiota would decrease tumor incidence in cancer susceptible populations.

## Abbreviations

A-T: Ataxia telangiectasia
RM: Restricted microbiota
CM: Conventional microbiota
KO: Knockout
UPLC: Ultra performance liquid chromatography
LC-MS: Liquid chromatography-mass spectrometry
TCA: Tricarboxylic acid
